# Connexin 43 Expression on Peripheral Blood Eosinophils: Role of Gap Junctions in Transendothelial Migration

**DOI:** 10.1155/2014/803257

**Published:** 2014-07-06

**Authors:** Harissios Vliagoftis, Cory Ebeling, Ramses Ilarraza, Salahaddin Mahmudi-Azer, Melanie Abel, Darryl Adamko, A. Dean Befus, Redwan Moqbel

**Affiliations:** Pulmonary Research Group, Department of Medicine, University of Alberta, 550 HMRC, Edmonton, AB, Canada T6G 2S2

## Abstract

Eosinophils circulate in the blood and are recruited in tissues during allergic inflammation. Gap junctions mediate direct communication between adjacent cells and may represent a new way of communication between immune cells distinct from communication through cytokines and chemokines. We characterized the expression of connexin (Cx)43 by eosinophils isolated from atopic individuals using RT-PCR, Western blotting, and confocal microscopy and studied the biological functions of gap junctions on eosinophils. The formation of functional gap junctions was evaluated measuring dye transfer using flow cytometry. The role of gap junctions on eosinophil transendothelial migration was studied using the inhibitor 18-a-glycyrrhetinic acid. Peripheral blood eosinophils express Cx43 mRNA and protein. Cx43 is localized not only in the cytoplasm but also on the plasma membrane. The membrane impermeable dye BCECF transferred from eosinophils to epithelial or endothelial cells following coculture in a dose and time dependent fashion. The gap junction inhibitors 18-a-glycyrrhetinic acid and octanol did not have a significant effect on dye transfer but reduced dye exit from eosinophils. The gap junction inhibitor 18-a-glycyrrhetinic acid inhibited eosinophil transendothelial migration in a dose dependent manner. Thus, eosinophils from atopic individuals express Cx43 constitutively and Cx43 may play an important role in eosinophil transendothelial migration and function in sites of inflammation.

## 1. Introduction

Eosinophils circulate in the blood and are recruited to peripheral tissues during allergic inflammation [1, 2]. Eosinophils are a rich source of chemokines, cytokines, and lipid mediators [3, 4], which can directly affect the activation status and survival of neighboring cells.

Cells of the immune system have been shown to communicate through gap junctions [5–7]. Gap junctions are channels that link the interior of two junction-forming cells and are formed by the end-to-end abutment of two hemichannels or connexons, which are comprised of six protein subunits called connexins (Cx) (reviewed in [8]). Gap junctions limit permeability to substances of less than 0.8–1 nm in molecular radius or less than 1 kDa. In this way, ions and small molecules such as second messenger molecules (cAMP, IP_3_) can pass from one communicating cell to the other in contrast to macromolecules, thus preserving intracellular integrity.

In peripheral tissues, eosinophils come in close contact to various cell types such as endothelial and epithelial cells. Endothelial and epithelial cells express connexins and form functional gap junctions with each other. Formation of gap junctions between eosinophils and tissue resident cells may provide another mechanism of interaction during inflammatory reactions. In this way activation of eosinophils through eosinophil specific stimuli could be transferred directly and specifically to immediately neighboring cells through gap junctions. This mechanism could lead to limited and specific activation of communicating cells compared to the activation obtained through the paracrine effects of released cytokines and chemokines. Gap-junction mediated interactions between immune cells and endothelial cells have been shown before [9, 10] and may have biological significance in inflammatory processes.

In this paper we characterized the expression of Cx43 by eosinophils and showed the formation of gap junctions between eosinophils and epithelial or endothelial cells. We also present evidence that gap junctions may play a role in eosinophil transendothelial migration.

## 2. Methods

### 2.1. Materials

Mouse monoclonal anti-Cx43 antibody (Zymed, South San Francisco, CA, USA); BCECF and BODIPY-conjugated goat anti-mouse IgG antibody (Molecular Probes, Eugene, OR, USA); DMEM, RPMI-1640, FBS, penicillin and streptomycin and L-glutamine (Biowhittaker, Walkersville, MD, USA); rabbit polyclonal anti-mouse HRP-conjugated antibody (Amersham Biosciences Corp., Baie D'Urfe, QC, Canada); nonfat dry milk and polyvinylidene difluoride (PVDF) membrane (Bio-Rad Laboratories Ltd., Mississauga, ON, Canada); MMLV reverse transcriptase, Taq polymerase, and oligo-dT primers (Gibco, Burlington, ON, Canada); anti-CD16 coated magnetic beads (Miltenyi Biotech Inc., Auburn, CA, USA); Costar clear Transwells for 12-well plates with 3 mm pores (Fisher Biosciences, Edmonton, AB, Canada); fibronectin coated Biocoat 12-well inserts with 3 mm pores (Becton Dickinson, Mississauga, ON, Canada); and octanol and 18-a-glycyrrhetinic acid and all other chemicals used (Sigma-Aldrich Canada Ltd., Oakville, ON, Canada) were purchased as shown.

### 2.2. Eosinophil Isolation

Eosinophils were isolated from peripheral blood of atopic individuals as previously described [11]. The study was approved by the University of Alberta Ethics Board. In brief, individuals with self-reported atopic disease and >3% eosinophils in the peripheral blood were recruited and 100 mL of blood was obtained by venopuncture. Granulocytes were isolated from the collected blood following gradient centrifugation over Ficoll. Granulocytes were then incubated with anti-CD16 coated magnetic beads to coat neutrophils and were applied on a magnetic column. Eosinophils were collected from the eluent and purity was analyzed using Kimura staining [12]. Eosinophil preparations used for our experiments had 97–99% purity by Kimura staining.

### 2.3. RT-PCR Analysis

RNA was isolated using Trizol. Two *μ*g RNA was reverse transcribed using MMLV and oligo-dT. PCR was performed using the following primers (synthesized by the DNA Services Laboratory, University of Alberta) for Cx43 (5′-TCACTTGGCGTGACTTCACTA-3′; 5′-CTGCTTCAAGTGCATGTCCAC-3′), Cx32 (5′-CAGGAGCCAGGTGTGGCAG-3′; 5′-AGCATCGGTCGCTCTTTTCAG-3′), and *β*2-microglobulin (5′-CTCGCGCTACTCTCTCTTTCTGG-3′; 5′-GCTTACATGTCTCGATCCGACTTAA-3′). Annealing temperature was 57°C for Cx43, 60°C for Cx32, and 58°C for *β*2-microglobulin. Forty cycles of amplification were performed in all cases except for *β*2-microglobulin where 22 cycles were performed. The amplified fragment sizes for *β*2-microglobulin, Cx43, and Cx32 were 335 bp, 439 bp, and 980 bp, respectively.

### 2.4. Western Blot Analysis

Cells were lysed in borate buffered saline with 1% Triton-X 100, 1 mM PMSF, 10 mg/mL aprotinin, 4 mg/mL leupeptin, and 10 mg/mL pepstatin, and protein concentration was measured with a colorimetric assay. Forty *μ*g of total cellular protein was separated on a 10% SDS-polyacrylamide gel and then transferred to a nitrocellulose membrane. The membranes were blocked with 5% dried milk and Western blot was performed with mouse anti-Cx43 monoclonal antibody (10 *μ*g/mL) and goat-anti-mouse-HRP secondary antibody (1/10,000). Proteins were visualized by enhanced chemiluminescence (ECL) on Kodak film.

### 2.5. Confocal Laser Scanning Microscopy (CLSM)

Cytospins of eosinophils were fixed in 2% paraformaldehyde in PBS for 10 min. Slides were washed five times in Tris-buffered saline, followed by incubation in a 2% human IgG blocking solution for 1 h. After a second wash step, mouse anti-Cx43 monoclonal antibody (20 *μ*g/mL) or isotype control (20 *μ*g/mL) was added for 1 h. Slides were subjected to a wash step again before BODIPY-conjugated goat anti-mouse antibody (50 *μ*g/mL) was added and cytospins were incubated for 1 h. Slides were examined under a Leica CLSM (Heidelberg, Germany). Images were stored on computer and transferred to Adobe Photoshop in advance of preparation of a composite image.

### 2.6. Cell Cultures

The human type II pneumocytes-like epithelial cell line A549 was acquired from the American Type Culture Collection (Rockville, MD, USA) and primary cultures of human microvascular endothelial cells isolated from the lung (HMVEC-L) were purchased from Clonetics (Walkersville, MD, USA). A549 cells were cultured in DMEM supplemented with 10% FBS, 4 mmol/L L-glutamine, and 100 mg/mL penicillin/streptomycin. HMVEC-L cells were grown in EBM-2 medium supplemented with human FGF, VEGF, IGF-1 and EGF, ascorbic acid, hydrocortisone, and 5% FBS as per distributor's instructions. These cells were used for up to four passages while they retained endothelial cell characteristics.

### 2.7. Cell Labeling with 2′,7′-Bis(2-carboxyethyl)-5(6)-carboxyfluorescein (BCECF)

Cells were washed in serum free medium and resuspended at 1 × 10^6^/mL. The membrane permeable dye BCECF-acetoxy methyl ester (BCECF-AM) (615 D) was added at a concentration of 1 *μ*M and the cells were incubated for 30 min at 37°C in a 5% CO_2_ incubator to allow dye to enter the cells where it is cleaved by esterases to the fluorescent, membrane impermeable acid form BCECF (520 D). Labeled cells are then washed in serum free medium and incubated for 30 min in medium supplemented with 10% FBS to allow any non-de-esterified dye to leave the cells, washed again in the same medium, and then used in dye transfer experiments.

### 2.8. Dye Transfer between Eosinophils and Epithelial or Endothelial Cells

To study the formation of functional gap junctions dye transfer experiments were performed as described, with minor modifications [13, 14]. Because eosinophils could be easily differentiated from epithelial or endothelial cells by their forward and side scatter characteristics we did not use a second fluorescent marker to differentiate the cell types following coculture. BCECF labeled eosinophils were cocultured in 12-well plates with 80–90% confluent cultures of A549 or HMVEC-L cells for the times indicated. To inhibit gap junction function we used octanol [15] and 18-a-glycyrrhetinic acid [16–18], as described. Octanol (up to 1 mM) or 18-a-glycyrrhetinic acid (up to 150 mM) was added to epithelial or endothelial cell cultures 15 min before initiation of coculture. Similar experiments were also performed between labeled and unlabeled HMVEC-L.

Following, coculture cells were detached with trypsin and analyzed on a FACScan flow cytometer (Becton Dickinson). Eosinophils were differentiated from epithelial or endothelial cells according to their forward and side scatter characteristics. To determine dye transfer, the fluorescent intensity of unlabeled cells following coculture with labeled cells was compared to the fluorescence intensity of cells incubated with the same number of unlabeled cells. Results are shown either as “% positive cells” or as “increase of relative fluorescence” between the two conditions.

In particular for Figure 4(a), unlabeled HMVEC-L cells were cocultured with labeled eosinophils for 1 h in the presence of 18-a-glycyrrhetinic acid or just diluent. The MFI of the HMVEC-L cells at the end of the coculture, as a consequence of dye transferred from labeled eosinophils, was then calculated with flow cytometry. We then expressed the MFI of cells cocultured in the presence of 18-a-glycyrrhetinic acid as a percent of the MFI of cells cocultured in the presence of diluent and subtracted this number from 100, since dye transfer would be expected to be lower in the presence of 18-a-glycyrrhetinic acid. This number was labeled as “decrease in mean fluorescence intensity” and is shown in Figure 4(a).

We then calculated the MFI of the labeled eosinophils or HMVEC-L cells in the same experiments in the presence of 18-a-glycyrrhetinic acid or diluent. We then calculated the “% change” in MFI between labeled cells cocultured in the presence of 18-a-glycyrrhetinic acid and labeled cells cocultured in the presence of diluent and this is the number shown in Figure 4(b). In all cases the MFI of the cells cultured in the presence of 18-a-glycyrrhetinic acid was higher than the MFI of those cultured in the presence of diluent.

### 2.9. Eosinophil Transmigration

HMVEC-L cells were grown on 12-well clear or fibronectin-coated inserts (3 mm pores) until confluent. Eosinophils or neutrophils (5 × 10^5^) were added to the upper chambers, and cells were allowed to transmigrate for 3 h towards eotaxin (50 ng/mL) or IL-8 (50 ng/mL) added in the lower chambers, respectively. Cells in the upper and lower chambers were then recovered, stained using a Kimura staining protocol [12], and counted. Results are shown as “total cells transmigrating ± SEM” or “% of added cells transmigrating ± SEM.”

### 2.10. Statistics

Student's* t*-test was used to analyze the results for statistical significance. A value of *P* < 0.05 was considered statistically significant.

## 3. Results

### 3.1. Cx43 Expression

**Figure 1:**
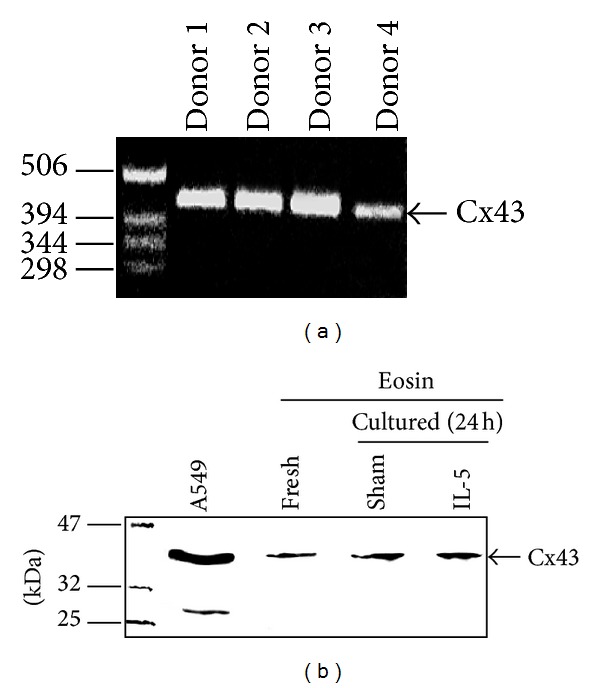
Cx43 expression by peripheral blood eosinophils: (a) RT-PCR for Cx43 expression in peripheral blood eosinophils from atopic donors. (b) Western blot analysis of Cx43 expression by freshly isolated eosinophils and eosinophils cultured for 24 h in the presence or absence of IL-5 (10 ng/mL).

Peripheral blood eosinophils expressed Cx43 mRNA (Figure 1(a)) but all individuals tested showed no expression of Cx32 (data not shown). We verified that peripheral blood eosinophils express Cx43 protein using Western blotting (Figure 1(b)). Incubation of eosinophils with IL-5 for up to 24 h did not change the level of expression of Cx43 protein (Figure 1(b)).

**Figure 2:**
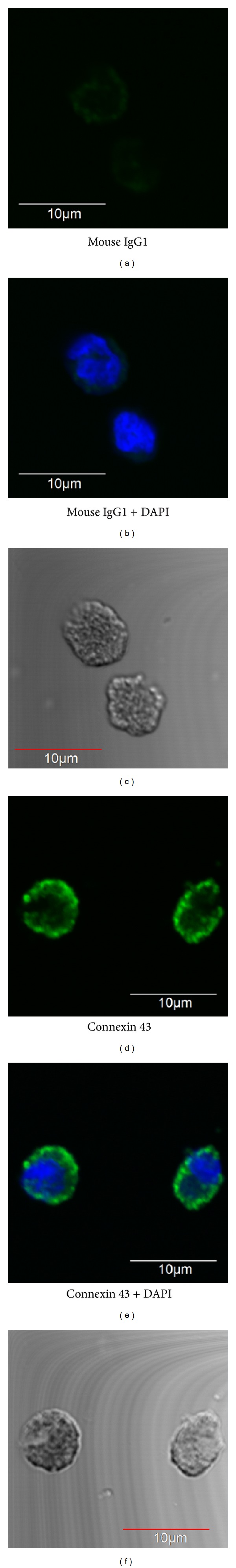
CLSM for Cx43 expression on freshly isolated human peripheral blood eosinophils. Staining with isotype control antibody (top panels) and mouse anti-Cx43 antibody (lower panels) is shown. Left panels show antibody staining alone and middle panels Cx43 staining along with DAPI to visualize the nuclei. The right panels show transmitted light images from the same slides.

To localize Cx43 protein in eosinophils we performed CLSM. All freshly isolated peripheral blood eosinophils expressed Cx43 (Figure 2) by CLSM. Staining in eosinophils was present on the plasma membrane as well as in the cytoplasm, but the cytoplasm staining was predominant. Therefore, eosinophils express Cx43 on the cytoplasmic membrane but they have a large pool of cytoplasmic Cx43.

### 3.2. Dye Transfer between Eosinophils and Other Cells

To study the ability of connexins to form functional gap junctions between eosinophils and other cell types we cocultured eosinophils with A549 human airway epithelial cells or HMVEC-L cells for 1–3 h. In different experiments eosinophils or epithelial/endothelial cells were loaded with the gap junction-permeable dye BCECF. Cells were cultured at a ratio of unlabeled to labeled cells of 1 : 2.

**Figure 3:**
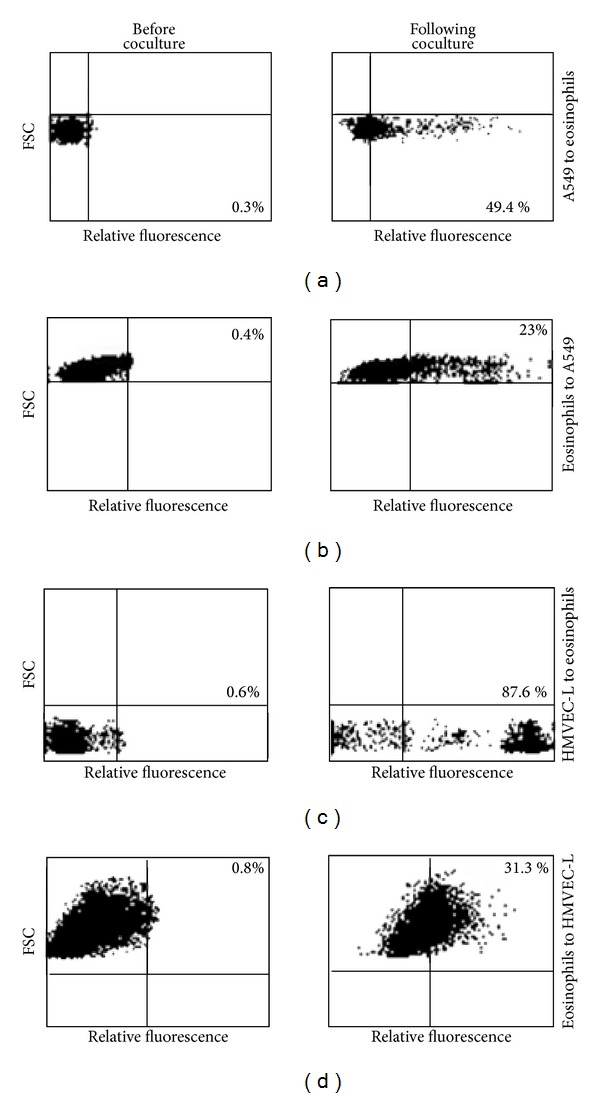
Dye transfer between eosinophils and epithelial/endothelial cells. Eosinophils were cocultured with A549 airway epithelial cells or HMVEC-L for 3 h. In each case one of the cell types was labeled and the other unlabeled. Unlabeled cells (eosinophils in (a) and (c), A549 cells in (b) and HMVEC-L in (d)) were gated and analyzed for evidence of dye transfer from the labeled cells. A representative experiment (from 4 to 6 experiments) is shown for each condition. Numbers in the graph indicate % of positive cells. Conditions: (a) transfer from labeled A549 cells to unlabeled eosinophils, (b) transfer from labeled eosinophils cells to unlabeled A549 cells, (c) transfer from labeled HMVEC-L cells to unlabeled eosinophils, and (d) transfer from labeled eosinophils to unlabeled HMVEC-L cells.

Dye transfer was observed between A549 and eosinophils in both directions (Figure 3(a) shows transfer from labeled A549 to unlabeled eosinophils and 3B transfer from labeled eosinophils to unlabeled A549 cells). Similarly dye transfer was demonstrated between eosinophils and HMVEC-L in both directions (Figure 3(c) shows transfer from labeled HMVEC-L to unlabeled eosinophils and 3D transfer from labeled eosinophils to unlabeled HMVEC-L). There was no evidence of cell death by the end of the coculture experiments. These experiments showed great variability in the numbers of unlabeled cells that became positive by the end of the experiment. For example, for dye transfer between unlabeled HMVEC-L cells and labeled eosinophils (Figure 3(d)), the percent of endothelial cells containing dye at the end of the experiment ranged from 15% up to 45% in different experiments. Similarly the MFI of endothelial cells containing dye transferred from eosinophils at the end of the experiment had a very wide range. This is the reason we only show a representative experiment out of the 5 experiments we performed. Among these conditions, dye transfer from HMVEC-L to eosinophils appeared to be most efficient (more than 85% or eosinophils became positive after coculture with calcein labeled HMVEC-L, Figure 3(c)). Dye transfer increased with increasing time of coculture and with increasing ratio of labeled to unlabeled cells (data not shown).

**Figure 4:**
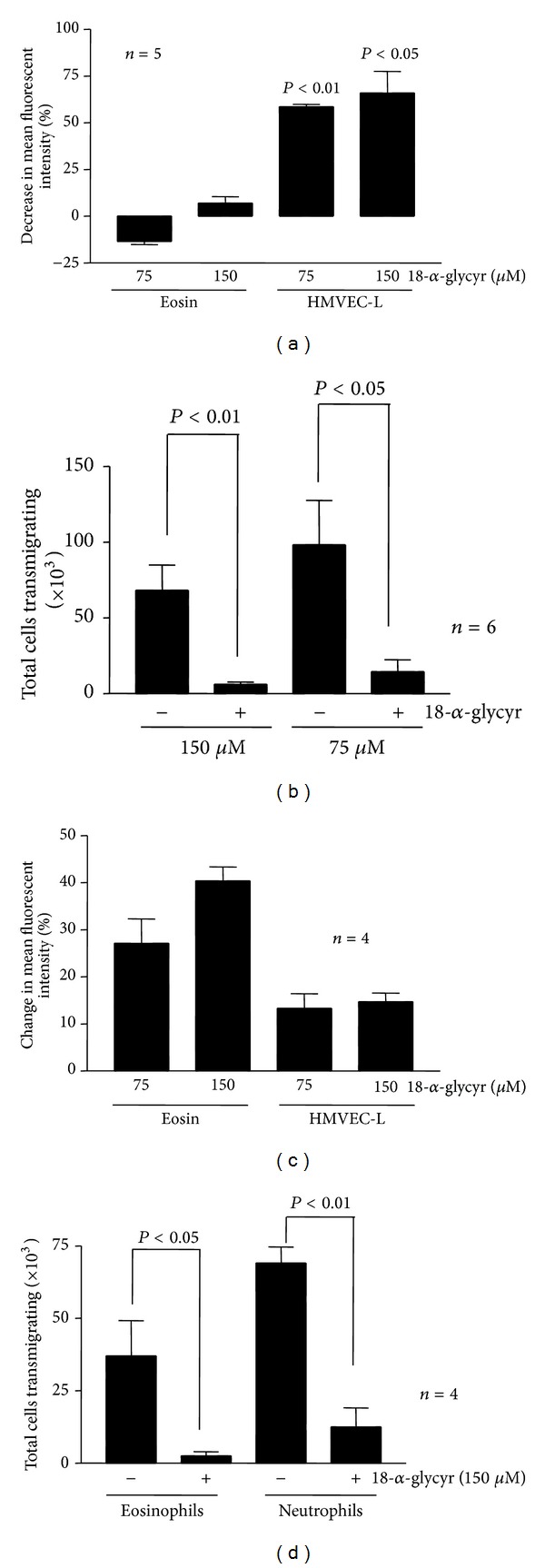
Effect of Cx inhibitors on dye transfer and transendothelial migration. (a) Unlabeled HMVEC-L cells were cocultured with labeled eosinophils (Eosin) or HMVEC-L (HMVEC-L). The graph shows % decrease in mean fluorescent intensity of unlabeled HMVEC-L in the presence of various concentrations of 18-a-glycyrrhetinic acid compared to mean fluorescent intensity in the absence of 18-a-glycyrrhetinic acid (*n* = 5). (b) Residual dye in labeled eosinophils or HMVEC-L following coculture with unlabeled HMVEC-L in the presence or absence of 18-a-glycyrrhetinic acid. The graph shows “% change mean fluorescent intensity (amount of dye)” in labeled eosinophils or HMVEC-L in the presence of various concentrations of 18-a-glycyrrhetinic acid compared to dye content in the absence of 18-a-glycyrrhetinic acid (*n* = 4). (c) Effect of various concentrations of 18-a-glycyrrhetinic acid on eosinophil transmigration through endothelial monolayers grown on transwells without fibronectin. (d) Effect of 18-a-glycyrrhetinic acid in transmigration of eosinophils or neutrophils through endothelial monolayers grown on transwells coated with FN.

To study whether this dye transfer was the result of gap junction-mediated communication between eosinophils and endothelial cells we used the gap junction inhibitor 18-a-glycyrrhetinic acid. We compared the ability of 18-a-glycyrrhetinic acid to affect dye transfer between eosinophils and endothelial cells with its effect on dye transfer between labeled and unlabeled endothelial cells. In both cases there was significant transfer of dye between labeled and unlabeled cells. Unlabeled HMVEC-L increased their fluorescence by 30–50-fold when cocultured with labeled HMVEC-L and only 3–10-fold when cocultured with labeled eosinophils. Preincubation of the cells for 15 min with 18-a-glycyrrhetinic acid decreased the amount of the dye transferred from labeled HMVEC-L to unlabeled HMVEC-L but had no effect on the transfer from labeled eosinophils to unlabeled HMVEC-L (Figure 4(a)). However, when the residual dye in labeled cells was analyzed, both HMVEC-L and eosinophils showed a lower magnitude of dye loss in the presence of 18-a-glycyrrhetinic acid (Figure 4(b)). Similar results were obtained using another inhibitor, octanol (data not shown).

### 3.3. Eosinophil Transendothelial Migration

There is evidence that gap junctions are important in regulating the transmigration of malignant cells through endothelial monolayers [13]. All eosinophils transmigrating through a monolayer of calcein-labeled HMVEC-L cells acquired significant amount of dye, presumably as a consequence of transfer via gap junctions (data not shown). Thus, to investigate the possible biological effects of gap junction formation between eosinophils and endothelial cells we studied the effects of inhibition of gap junctions on eosinophil transendothelial migration. The numbers of transmigrating eosinophils were variable in different experiments ranging from 6 to 26% (15.4 ± 3.4%, *n* = 6) of the totals cells that were added to the upper chamber. The gap junction inhibitor 18-a-glycyrrhetinic acid inhibited the number of migrating eosinophils in both concentrations studied (Figure 4(c)). The inhibitor did not affect cell viability (data not shown).

Similar experiments were repeated using transwells coated with FN to grow the endothelial cells. In this case again 18-a-glycyrrhetinic acid inhibited the transmigration of eosinophils (Figure 4(d)). We also tested the effect of 18-a-glycyrrhetinic acid on neutrophil migration through endothelial cells grown in FN-coated transwells. Neutrophil transmigration was also inhibited by 18-a-glycyrrhetinic acid (Figure 4(d)).

## 4. Discussion

We have shown that human peripheral blood eosinophils from atopic individuals express Cx43 but not Cx32. Cx43 was expressed on the cell membrane and also diffusely in the cytoplasm of freshly isolated peripheral blood eosinophils. Dye transfer experiments showed evidence of functional gap junction formation between eosinophils and epithelial or endothelial cells. Inhibitors of gap junction function showed a role for gap junctions in transmigration of eosinophils and neutrophils through endothelial monolayers.

Connexins are a family of more than 20 proteins [19], at least some of which are expressed by immune cells [9, 16, 20–22]. Cx43 is the most widely studied connexin in the immune system but at least mast cells [20] and lymphocytes [21] express other connexins. Connexins have been shown to participate in various immune processes. Connexins play a role in maturation [22] and activation [23] of immune cells. Gap junctions participate in T cell proliferation and clonal expansion [24], in cross-presentation and cross-priming [25, 26], and in interactions of T regulatory cells with dendritic cells [27] and effector T cells [28]. All these relatively new observations support an important role of gap junctions in the development of immune responses. In addition, communications between immune and nonimmune cells through gap junctions may offer an alternative pathway that mediates important effects of the immune system on tissue homeostasis and/or inflammation.

Gap junction formation has been observed between immature eosinophils in the bone marrow [29], which may indicate that gap junctions contribute to eosinophil maturation. Our data indicate that, following tissue localization, eosinophils might form gap junctions with tissue resident cells to allow them more efficient communication with their microenvironment. Connexins, and especially Cx43, have also been associated with protection from apoptosis [19]. These functions of connexins may be very important for eosinophils and other inflammatory cells.

The two inhibitors of gap junctions we used did not show clear evidence of inhibition of dye transfer between eosinophils and epithelial/endothelial cells. However, this is to our knowledge the main pathway that could mediate direct transfer of a membrane impermeable dye between eosinophils and epithelial/endothelial cells. Different connexin molecules can form gap junctions with varying conductance properties and differential sensitivity to gap junction inhibitors [19]. It is therefore possible that eosinophils express, in addition to Cx43, other connexins that form gap junctions that are not sensitive to the inhibitors we used.

Connexins, in the form of open hemichannels, are also functional between the intracellular and extracellular environments [19] and may regulate cell volume and composition of intracellular environment or mediate ATP [30, 31] and NAD^+^ [32] release in the extracellular environment. Our data using gap junction inhibitors showed diminished loss of membrane impermeable dye from eosinophils (Figure 4). These data may be the result of connexins forming hemichannels that mediate dye loss and the inhibition of these hemichannels by the gap junction inhibitors we used.

It is important to define the cell types that communicate with eosinophils through gap junctions. Endothelial cells could be candidate partners for gap junction formation with eosinophils and also with other immune cells. Immune cell transmigration through the endothelium is probably a more complicated process than currently understood. Gap junctions between transmigrating immune cells and endothelial cells could facilitate transfer of important stimuli that initiate events leading to immune cell transmigration.

Our data suggest gap junction involvement in eosinophil transendothelial migration. Recent observations have also implicated gap junction functions in neutrophil transendothelial migration [16]. However, some of our observations are inconsistent; for example, the gap junction inhibitor 18-a-glycyrrhetinic acid had a pronounced effect on eosinophil transendothelial migration but did not affect dye transfer between eosinophils and endothelial cells. One possibility is that 18-a-glycyrrhetinic acid affects eosinophil transmigration by inhibiting exclusively gap junctions between adjacent endothelial cells. This hypothesis would indicate that gap junction-mediated endothelial cell communication is required for eosinophil transmigration. Alternatively, it could also suggest that eosinophil connexins form hemichannels that communicate with the extracellular environment, which may play a role in eosinophil transmigration. Our observation that 18-a-glycyrrhetinic acid also inhibited neutrophil transendothelial migration is not in agreement with recently published data [16]. However, in that study the authors used a different endothelial cell source (umbilical vein endothelial cells—HUVEC), a different stimulus for transmigration (fMLP), and inhibited Cx43 with a synthetic peptide, rather than inhibiting several connexins with a chemical inhibitor.

In conclusion we have presented biochemical and functional evidence that eosinophils express connexins, which may facilitate transfer of small molecules to epithelial and endothelial cells through gap junctions. The physiological relevance of gap junctions between eosinophils and epithelial or endothelial cells remains to be investigated. Our evidence suggests that gap junctions may play a role in eosinophil transendothelial migration. These heterologous gap junctions between immune and nonimmune cells might represent an underrecognized pathway for communication between eosinophils and cells in the local microenvironment in sites of inflammation.

## 5. Conclusions

Eosinophils express Cx43 that is functional and allows communication of eosinophils with other cells. This communication may be very important for eosinophil transmigration through the endothelial monolayer and tissue infiltration.

## Abbreviations

18-a-glycyr: 18-a-Glycyrrhetinic acid
BCECF: 2′,7′-Bis(2-carboxyethyl)-5(6)-carboxyfluorescein
Cx: Connexin
Cx43: Connexin 43
Cx32: Connexin 32
CLSM: Confocal laser scanning microscopy
HMVEC-L: Human microvascular endothelial cells from lung
Treg: T regulatory cells.
